# Semi-Automatic Tracking of Laser Speckle Contrast Images of Microcirculation in Diabetic Foot Ulcers

**DOI:** 10.3390/diagnostics10121054

**Published:** 2020-12-06

**Authors:** Onno A. Mennes, Mark Selles, Jaap J. van Netten, Jeff G. van Baal, Wiendelt Steenbergen, Riemer H. J. A. Slart

**Affiliations:** 1 Biomedical Photonic Imaging, University of Twente, 7500 AE Enschede, The Netherlands; o.a.mennes@utwente.nl (O.A.M.); r.h.j.a.slart@umcg.nl (R.H.J.A.S.); 2 Ziekenhuisgroep Twente, ZGT Academy, 7609 PP Almelo, The Netherlands; j.vbaal@zgt.nl; 3 Technical Medicine, University of Twente, 7500 AE Enschede, The Netherlands; m.selles@student.utwente.nl; 4 Amsterdam Movement Sciences, Department of Rehabilitation, University of Amsterdam, Amsterdam UMC, 1105 AZ Amsterdam, The Netherlands; j.j.vannetten@amsterdamumc.nl; 5 Institute of Health and Biomedical Innovation, School of Clinical Sciences, Queensland University of Technology, Brisbane, QLD 4000, Australia; 6 School of Medicine, Cardiff University, Wales CF10 3AT, UK; 7 Department of Nuclear Medicine and Molecular Imaging, Medical Imaging Center, University Medical Center Groningen, 9713 GZ Groningen, The Netherlands

**Keywords:** Laser Speckle Contrast Imaging, diabetic foot ulceration, microcirculation, tracking algorithm

## Abstract

Foot ulcers are a severe complication of diabetes mellitus. Assessment of the vascular status of diabetic foot ulcers with Laser Speckle Contrast Imaging (LSCI) is a promising approach for diagnosis and prognosis. However, manual assessment during analysis of LSCI limits clinical applicability. Our aim was to develop and validate a fast and robust tracking algorithm for semi-automatic analysis of LSCI data. The feet of 33 participants with diabetic foot ulcers were recorded with LSCI, including at baseline, during the Post-Occlusive Reactive Hyperemia (PORH) test, and during the Buerger’s test. Different regions of interest (ROIs) were used to measure microcirculation in different areas of the foot. A tracking algorithm was developed in MATLAB to reposition the ROIs in the LSCI scans. Manual- and algorithm-tracking of all recordings were compared by calculating the Intraclass Correlation Coefficient (ICC). The algorithm was faster in comparison with the manual approach (90 s vs. 15 min). Agreement between manual- and algorithm-tracking was good to excellent during baseline (ICC = 0.896–0.984; *p* < 0.001), the PORH test (ICC = 0.790–0.960; *p* < 0.001), and the Buerger’s test (ICC = 0.851–0.978; *p* < 0.001), resulting in a tracking algorithm that delivers assessment of LSCI in diabetic foot ulcers with results comparable to a labor-intensive manual approach, but with a 10-fold workload reduction.

## 1. Introduction

The incidence of diabetes mellitus increases worldwide, followed by an increasing number of related complications such as diabetic foot disease [1]. It is estimated that diabetic foot disease ranks in the top ten of global burden of diseases [2]. The most common aspect of this disease are diabetic foot ulcers, which are related to peripheral neuropathy and peripheral artery disease (PAD) [3]. Due to peripheral neuropathy, tissue damage to the foot can go unnoticed. At the same time, PAD and capillary dysfunction cause ischemia, and impaired wound healing if an ulcer develops [4,5].

Treatment of diabetic foot ulcers relies on five major factors, including: relief of pressure, and protection and care of the ulcer; restoration of skin perfusion; treatment of infection; metabolic control; and treatment of co-morbidities [6]. To determine the need of restoration of skin perfusion, it is essential to determine the vascular status of a patient. Based on this assessment, treatment decisions such as the need for revascularization are taken. However, this vascular assessment is one of the biggest challenges in the treatment of diabetic foot ulcers [6].

The clinical standard for vascular assessment consists primarily of non-invasive assessment of blood pressure (e.g., by determining the ankle–brachial index (ABI), toe–brachial index (TBI), or transcutaneous oxygen pressure (TcPO2)). However, non-invasive blood pressure measurements fail to adequately assess microcirculation in the area of the ulcer. ABI and TBI only assess macrocirculation in the lower extremities, while with TcPO2 the microcirculation is assessed locally, but not necessarily in or adjacent to the ulcer, and it can be influenced by other factors such as edema or infection [7]. Furthermore, the reliability of non-invasive assessment of blood pressure in people with diabetes is questionable as vascular calcification stiffens the arterial wall, making arteries poorly compressible [8].

Novel optical imaging techniques such as Laser Speckle Contrast Imaging (LSCI) are promising for the assessment of microcirculation, and not yet applied as clinical routine. LSCI provides real-time, non-contact imaging of the superficial microcirculation of foot tissue, and images a large tissue area [9]. Therefore, LSCI could be an eligible tool for the assessment of microcirculation in patients with diabetic foot ulcers [10,11].

LSCI is a technique that can image and estimate blood flow in tissue down to a depth of 1–1.5 mm [10]. When coherent light is diffusely backscattered by a medium, it forms an interference pattern also called a speckle pattern. Moving particles in the illuminated medium will cause temporal fluctuations in the speckle pattern. The presence of more and faster-moving particles results in more and faster fluctuations in the speckle pattern, and causes a blurred speckle pattern image when imaged with an exposure time larger than the timescale of the speckle fluctuations [12,13,14]. By quantifying the contrast of the speckles, an indication of the concentration of moving particles (which are predominantly red blood cells in the case of tissue imaging) and their speed can be made [12]. Quantification of the movement of the red blood cells in the skin results in an estimation of perfusion. LSCI can therefore complement the currently used non-invasive blood pressure measurements to assess the level of ischemia in diabetic foot ulcers, for example directly at or adjacent to the ulcer location. Furthermore, it can be performed during stress tests that provide additional clinical information, such as the Post-Occlusive Reactive Hyperaemia (PORH) test, where the peak blood flow is measured after an occlusion of the ankle, or the Buerger’s test, where the leg is raised to reduce blood flow in the foot for 30–60 s [11,15,16].

When LSCI is applied to assess diabetic foot ulcers, different regions of interest (ROIs) can be assessed simultaneously. This is useful, since both the ulcer and the ulcer edge are of particular interest, and also allows for analysis of multiple ulcers on one foot [10]. Currently, ROIs have to be drawn manually to analyze the measurements. This is time consuming and limits the clinical applicability of LSCI. An algorithm that assists in positioning the ROI during the entire measurement, and that performs despite the presence of foot or leg movements, may help to overcome this drawback.

The aim of this study was to (1) develop, and (2) validate a fast and robust tracking algorithm for semi-automatic analysis of LSCI data, to improve the clinical applicability of LSCI.

## 2. Materials and Methods

### 2.1. Data Acquisition

The clinical dataset was obtained as part of a larger study [17]. This study was approved by a registered medical ethics committee and the study was registered in the Dutch trial register (NTR5116; 25-03-2015). Thirty-three patients with a diabetic foot ulcer participated. LSCI measurements of each ulcerated foot were performed by two operators (operator A and operator B), resulting in a total of 66 LSCI recordings. The recordings were obtained using a PeriCam PSI (Perimed AB, Stockholm, Sweden). Before each use, the system was calibrated. During the measurements, both a PORH test and Buerger’s test were performed to amplify differences in perfusion between (critical-)ischemic and non-ischemic patients [11,15,16].

**Figure 1:**
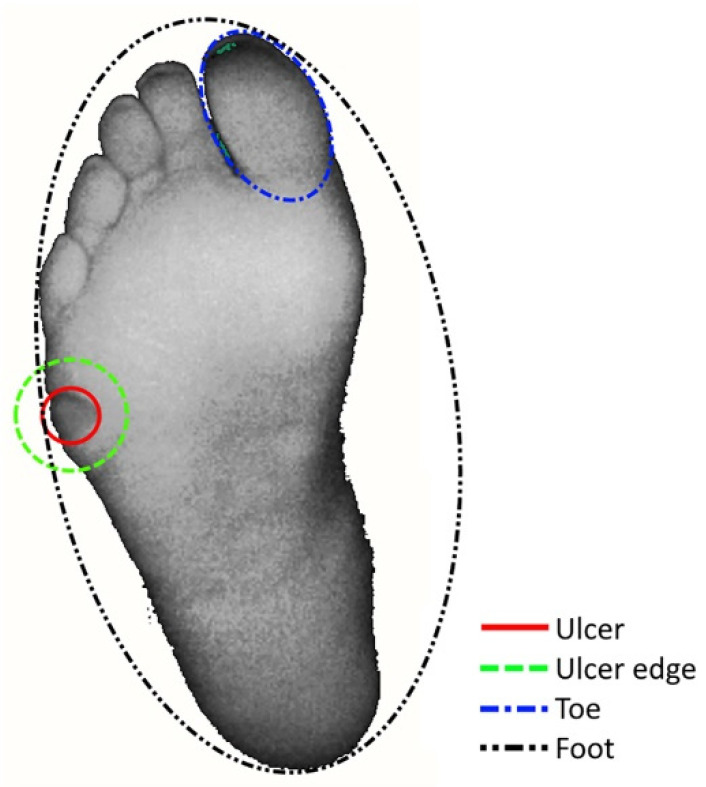
Placements of four Regions of Interest (ROIs) of Laser Speckle Contrast Imaging scans; (1) the ulcer, (2) the ulcer edge, (3) the toe (hallux or digitus II), and (4) the entire foot (in this case the plantar side).

**Figure 2:**
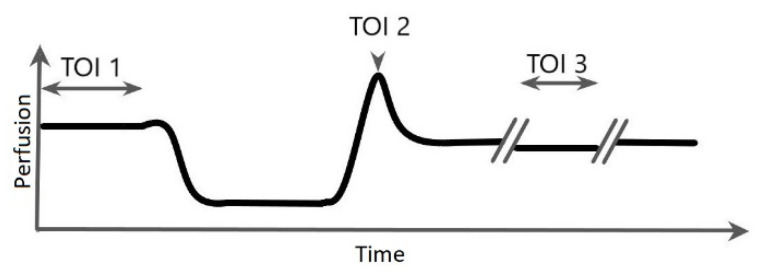
Placements of Timespans of Interest (TOI) of Laser Speckle Contrast Imaging scans. TOI 1: baseline, TOI 2: Post-Occlusive Reactive Hyperaemia test, peak perfusion after reperfusion. TOI 3: Buerger’s test. //: Short break in scan to reposition the foot before and after the Buerger’s test.

Four ellipse-shaped Regions of Interest (ROIs) were selected based on the greyscale intensity image of the LSCI scan, at three Timespans of Interest (TOIs). The ROIs included: (1) the ulcer, (2) the ulcer edge, (3) the toe (hallux or, when the hallux was amputated, digitus II), and (4) the entire plantar or dorsal side of the foot (the side where the ulcer was located was measured). Pixels in the background of the scan were excluded from the dataset and did not influence ROI placement or calculations of the microcirculation (Figure 1). The TOIs included: (1) baseline, (2) the moment of peak perfusion during the PORH test, and (3) the Buerger’s test (Figure 2). In the case that no well-defined peak perfusion was found during the PORH test, the maximum measured value when the perfusion stops increasing was used to define TOI 2. All ROIs were manually positioned for the first frame.

In manual tracking, ROIs were manually repositioned for each frame during the scan, to compensate for movement of the foot during measurement. For algorithm tracking, the ROIs from the first frame were used to automatically reposition the ROIs during the rest of the scan when required. A second set of ROIs was drawn before the Buerger’s test and automatically used for this TOI. The shape of the foot was detected using Canny edge detection [18] as implemented in MATLAB’s Image Processing Toolbox [19] for all frames of the recording. Mean perfusion values for each ROI during each TOI were calculated and used for data analysis, and were computed with Pimsoft software (version 1.5; Perimed AB, Stockholm, Sweden).

### 2.2. Algorithm Development

An algorithm was developed with custom-written code in MATLAB (The MathWorks Inc.; Natic, MA, USA). Following manual assessment of ROIs in the first frame, the ROIs in frames [2, n] were computed using the Iterative Closest Point (ICP) algorithm [20] as implemented in MATLAB’s Computer Vision System Toolbox [21]. ICP computes the transformation matrix between two images to align two images. The transformation matrix consists of a rotational and a translational component.

**Figure 3:**
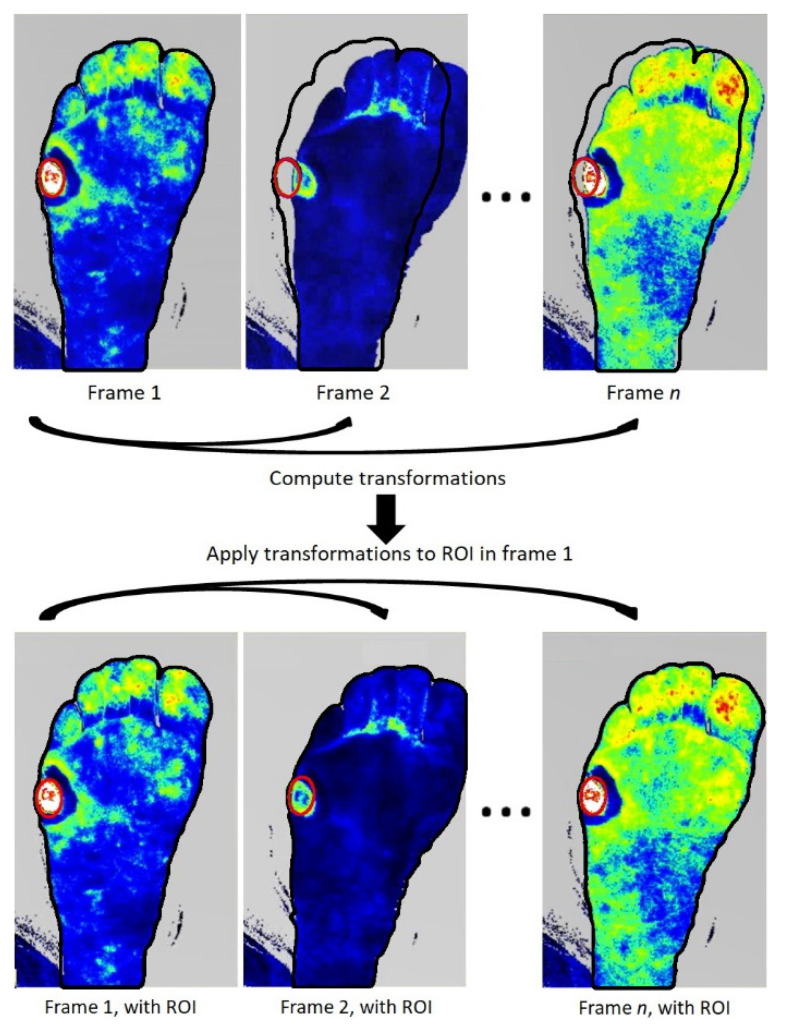
Principle of the region of interest (ROI) tracking. The transformation from frame 1 towards the other frames is computed based on the shape of the foot in both frames. The ROI in the frames [2, n] is computed by application of the transformation from frame 1 to frame n on the initial ROI. If, as shown, the foot moves to right, the ROI follows correct placement at the ulcer location.

Likewise, the transformation matrices of frame 1 to frames [2, n] were computed based on the shape of the foot, resulting in n-1 transformations. Application of the corresponding transformation matrices to the ROIs as defined in frame 1 resulted in the ROIs in the frames [2, n] (Figure 3).

**Figure 4:**
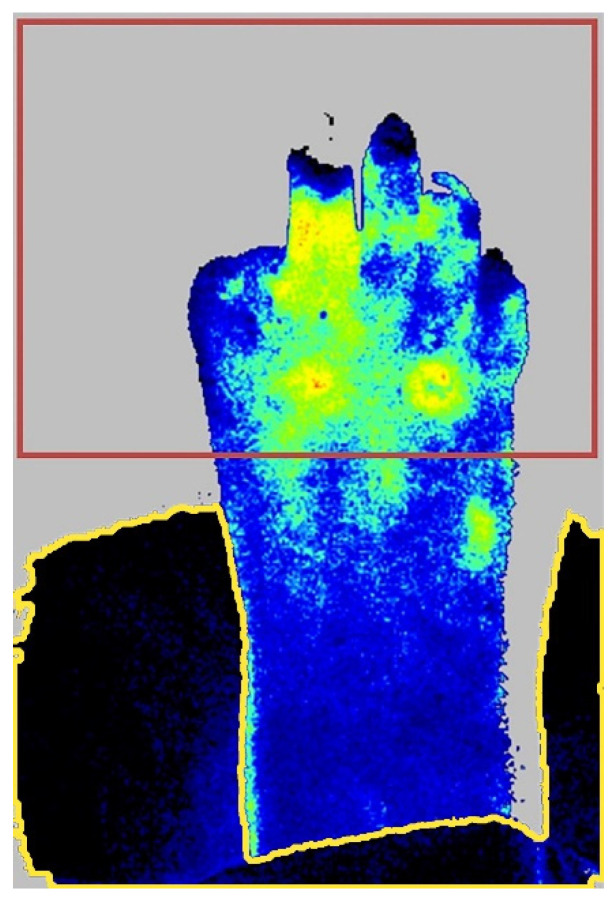
Example of a Laser Speckle Contrast Imaging recording with static artefacts. The red box defines the selected area of the image that was used for ROI tracking. Static artefacts (e.g., the examination bench and pressure band) are highlighted by the yellow outline and were ignored when calculating the transformation matrices.

To prevent erroneous calculations of ROIs due to static artefacts in the background (such as the examination bench), the region without static artefacts was once selected manually in frame 1 of the recording (Figure 4). Consequently, the region without static artefacts was used to calculate the transformation matrices and compute the ROIs in the frames [2, n].

**Figure 5:**
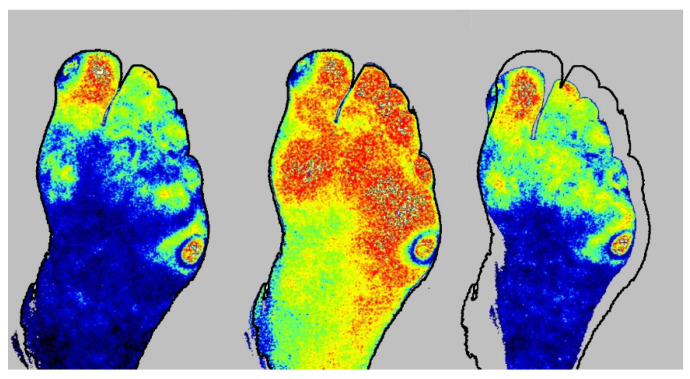
ROI tracking during baseline measurement (**left**), Post-Occlusive Reactive Hyperaemia test (**middle**), and Buerger’s test (**right**). Here, the ROI during Buerger’s test was erroneous, illustrating the need for redrawing the ROIs.

Because of the repositioning of the foot during the Buerger’s test, the shape of the imaged foot changed, with the foot becoming a non-rigid object (Figure 5). Therefore, a second set of ROIs was manually drawn for the first frame of the Buerger’s test. The transformation matrices for the Buerger’s test frames were reset, and new transformations and ROI repositioning were used in this TOI.

The perfusion was calculated in MATLAB, identically to the perfusion calculations as done by the Pimsoft software (Perimed AB, Stockholm, Sweden). The equations as stated below were provided by Perimed. The intensity and variance were used to calculate the perfusion in the ROIs for all TOIs. First, the mean intensity 〈*I*〉 and the standard deviation σ in all four ROIs were calculated for TOI 1, TOI 2, and TOI 3.

Then, the mean contrast was computed by [22]

(1) $$K=\beta \frac{\sigma }{\langle I\rangle }$$

with *β* as the coherence factor. The coherence factor is instrument-specific and dependent on calibration and ensures that *K* = 1 for static objects. The coherence factor was obtained by exporting the recordings to MATLAB.

From the mean contrast the mean perfusion was calculated as

(2) $$P=k(\frac{1}{K}-1)$$

with k the signal gain. Similar to the coherence factor β, the signal gain is also instrument-specific and ensures that an instrument, after calibration, measures the same perfusion value when measuring the same tissue. The calibration for both the signal gain (k) and coherence factor (β) was performed by measuring a zero-perfusion area and a colloidal suspension of polystyrene particles to set the LSCI values on 0 ± 5 perfusion units (PU) and 250 ± 5 PU respectively.

### 2.3. Algorithm Validation

**Figure 6:**
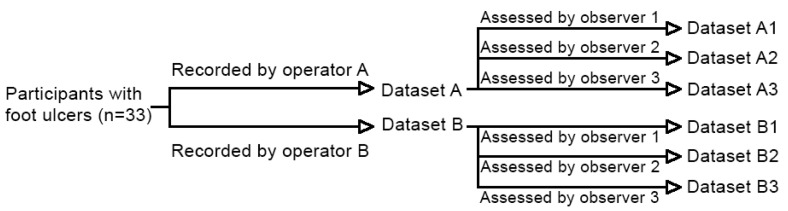
Schematic overview of datasets.

All 66 LSCI scans were recorded by two operators (operators A and B). All scans were manually analyzed by two assessors (assessor 1 [the same as operator A] and assessor 2) and all scans were analyzed by the tracking algorithm. Time required for each of the analyses (manual or algorithm) was measured by performing 10 measurements and calculating the average time needed for the manual input. Performing these assessments resulted in the mean perfusion for all four ROIs during all three TOIs. By subjecting the two datasets of operator A and B to the analyses of the two different assessors and the algorithm, this process resulted in a total of six datasets (Figure 6).

To determine the intra-assessor variation, Intraclass Correlation Coefficient (ICC) with a two-way random effects model was calculated between the different datasets. The definition used for ICC testing was absolute agreement, where values less than 0.5 are indicative of poor reliability, values between 0.5 and 0.75 are moderate, between 0.75 and 0.9 are good, and greater than 0.90 are indicative of excellent reliability [23]. Statistical analyses were performed using SPSS version 23.0 (SPSS Inc. Chicago, IL, USA).

## 3. Results

### 3.1. Algorithm Performance

The tracking algorithm was able to analyze all LSCI scans. No additional ROI drawing apart from in the first frame of the baseline measurement and Buerger’s test was needed. The labor time of manual selection of all ROIs in both frames was approximately 90 s and the mean analyses duration of the algorithm was 185 s. This was faster than the manual assessment of an entire scan, that took on average 15 min per scan.

**Figure 7:**
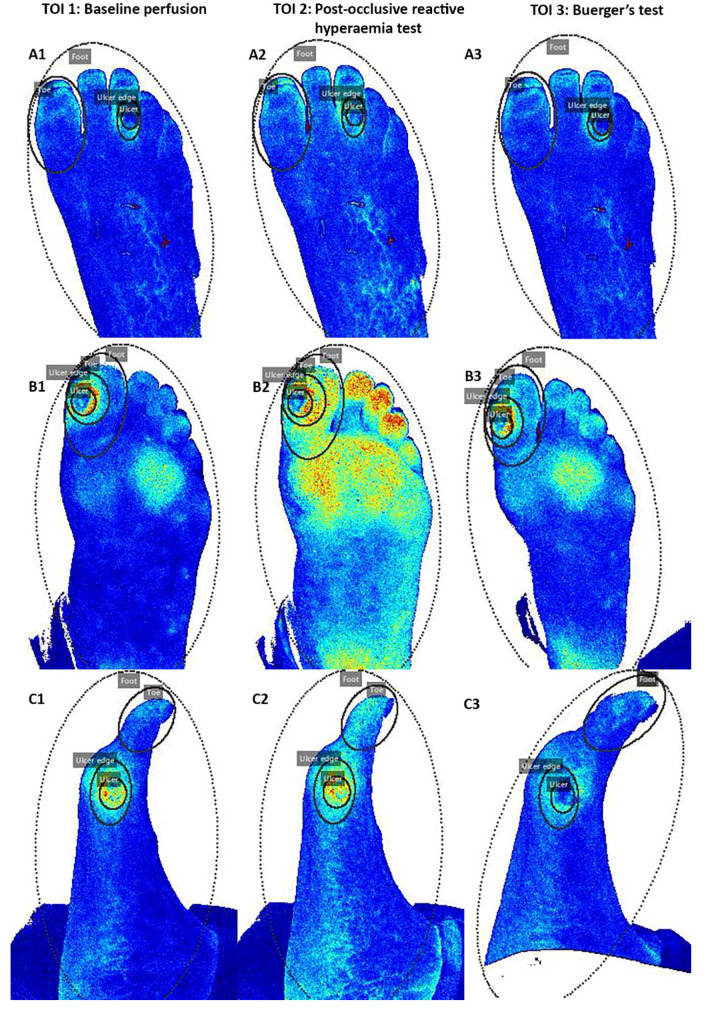
ROI tracking during baseline measurement (**left**), Post-Occlusive Reactive Hyperaemia Test (**middle**), and Buerger’s test (**right**) for three different patients (**A** (plantar view), **B** (plantar view), **C** (medial view)). The ROI during Buerger’s test was manually repositioned for the first frame of the test.

The algorithm was capable of repositioning the ROIs in all scans. The algorithm correctly repositioned the ROIs in the majority of the cases (Figure 7, videos available in Supplementary Materials). The quality of the repositioning and the analyses of the LSCI scan varied within and between different scans. In 5.1% (40 of 792) of all ROI placements, some suboptimal ROIs were drawn resulting in a measured PU value 10% higher or lower than measured during the manual approach. The frequency of those suboptimal ROIs was comparable between baseline (n = 15, 5.7%), PORH (n = 15, 5.7%), and Buerger’s test (n = 13, 4.9%).

### 3.2. Algorithm Validation

**Figure 8:**
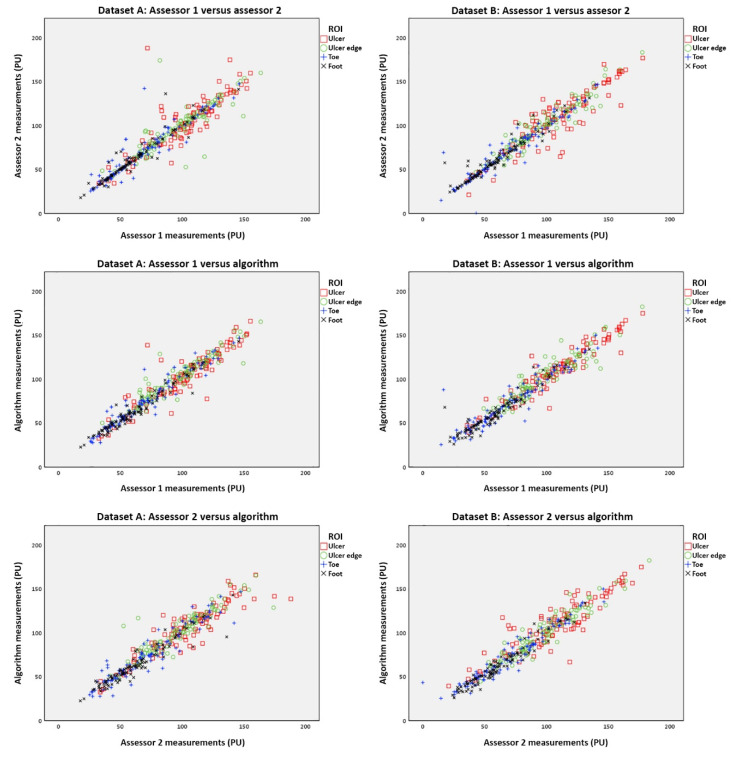
Scatterplots of the measurements between assessors and the algorithm. Perfusions are shown in Perfusion Units (PU) for all scans during baseline measurements and different stress tests. Recordings were obtained with Laser Speckle Contrast Imaging by operator A (Dataset A) and operator B (Dataset B), and analyzed by two assessors and the algorithm. Assessors 1 and 2 and the algorithm were compared to each other for different regions of interest (ROIs).

The ICCs were good to excellent for all comparisons between manual and algorithm tracking, and between the two manual trackings (Figure 8, Table 1).

In dataset A, the ICCs between both assessors and the algorithm were good to excellent (ICC = 0.790–0.978) and higher than the ICCs between both human assessors (ICC = 0.628–0.988). Baseline ICCs (ICC = 0.896–0.975) were comparable to stress-test ICCs (POHR: ICC = 0.790–0.952; Buerger’s test: ICC = 0.894–0.978) (Table 1).

In dataset B, the ICCs between both assessors and the algorithm were good to excellent (ICC = 0.851–0.984) and comparable to the ICCs between both human assessors (ICC = 0.865–0.992). Baseline ICCs (ICC = 0.912–0.984) were higher compared to stress-test ICCs (POHR: ICC = 0.865–0.960; Buerger’s test: ICC = 0.851–0.953) (Table 1).

When no additional ROIs were drawn on the first frame of the Buerger’s test, the ICCs for the Buerger’s test decreased, particularly against the algorithm (range: 0.532–0.980).

## 4. Discussion

We aimed to develop and validate a fast and robust tracking algorithm for analysis of LSCI data of diabetic foot ulcers, to improve clinical applicability of LSCI in vascular assessment in diabetic foot disease. We developed an algorithm that required minimal manual input and was able to process all LSCI data. This algorithm can do this with an approximately 10-fold workload reduction compared to the current manual approach with software from the manufacturer, and demonstrated good to excellent agreement with this current standard approach. This shows that the algorithm achieves human-like performance for the assessment of LSCI data of diabetic foot disease. Currently the time-consuming analysis of LSCI data for the assessment of diabetic foot ulcers limits its clinical applicability; the developed algorithm is fast enough to overcome this.

While we advocate implementation of the algorithm in clinical practice, it should be noted that some of the ROIs were repositioned suboptimally by the algorithm. Although the incidence of these suboptimal ROIs was low, inspection of the ROIs yielded by the algorithm is encouraged to examine if the ROIs are repositioned correctly. Besides rigid registration, several nonrigid registration algorithms have been developed. B-spline based nonrigid registration has shown to be successful in infrared thermography of the diabetic foot [24]. This approach might reduce the suboptimal ROIs, and might also eliminate the required redefinition of ROIs at the first frame of the Buerger’s test, further minimizing the need for human input. On the other hand, non-rigid registration is generally more time-consuming, prolonging the duration of analysis as the registration has to be performed for all frames of the LSCI recording. This needs to be investigated in future research.

The optimum analysis method of LSCI data would be a fully automated one, by automatic detection of the ulcer in the first frame. This would eliminate erroneous measurements due to human intervention and would potentially also speed up the process of analysis even further. Promising results have been shown using deep learning for the automatic segmentation of diabetic foot ulcers [25,26]. However, these results were achieved using RGB images, not greyscale LSCI intensity data. Either automatic detection of ulcers in LSCI perfusion data or mapping of the ulcer segmentations from RGB images to the LSCI data should be developed to make this automatic segmentation approach useful for ulcer assessment using LSCI. We think it would be easier to use RGB images of the foot instead of the greyscale intensity data or the measured perfusion data, because using RGB images allows other factors, such as the color (for example, redness) of the skin, to also be taken into account. Automatic detection of ulcers might also facilitate more accurate assessment of the ulcer and ulcer edge, following their real contours. We used ellipse-shaped ROIs, whereas automatic detection can draw along the ulcer and along the edge of the ulcer.

There are some limitations to this study. First of all, the semi-automatic algorithm still requires human input, at the start of the LSCI recording and at the first frame of the Buerger’s test. During this study, one researcher provided the input that was needed for the algorithm. Future research should investigate the inter-rater reliability when different researchers or clinicians provide the input for the algorithm. Second, in this study, we only investigated the mean PU values calculated for each ROI and compared those values with each other to measure the ICC, because this is clinically most relevant. Given the complex pattern and perfusion hotspots in and around the ulcer, it makes sense that the ROIs were positioned correctly if the measured PU was comparable with the human measurements. However, the exact location and size of the ROI placements were not compared with each other. This could be interesting for further research. Third, a possible learning curve of the assessor to analyze the data and position the different ROIs was not taken into account in this study. The time needed for the manual placement of the ROIs was based on the last measured patients. This time might be further reduced if the assessor has more experience with the data and the positioning of different ROIs in the LSCI scans.

LSCI is currently being investigated in diabetic foot disease as well as several other medical fields such as rheumatology, dermatology, ophthalmology, neurology, and gastro-intestinal surgery [14]. Also, in these fields, ROIs at several timepoints or timespans are used for assessment of perfusion and tracking of the ROIs might be useful. Application of the algorithm needs to be assessed in those fields. However, it should be noted that our algorithm is not useful in all fields. For instance, ROI tracking for LSCI assessment of cerebral blood flow requires different registration approaches [27]. As every application of LSCI may require a different approach to address movement of ROIs, this should be evaluated per application.

## 5. Conclusions

The developed tracking algorithm for analysis of LSCI data of diabetic foot ulcers has a good to excellent inter-rater reliability in comparison to the current standard of manual assessment. The algorithm shows a 10-fold workload reduction compared the manual approach, and may improve clinical applicability of LSCI for the assessment of diabetic foot disease.

## Figures and Tables

**Table 1:** Intraclass Correlation Coefficients (ICC) of assessment of Laser Speckle Contrast Imaging with two Regions of Interest placements before baseline and Buerger’s test measurements.

Characteristics		Dataset A			Dataset B	
	Assessor 1 vs. 2	Assessor 1 vs. Alg.	Assessor 2 vs. Alg.	Assessor 1 vs. 2	Assessor 1 vs. Alg.	Assessor 2 vs. Alg.
**Baseline**						
Ulcer	0.936	0.968	0.953	0.950	0.984	0.954
Ulcer edge	0.936	0.975	0.896	0.966	0.969	0.963
Toe	0.983	0.953	0.942	0.992	0.963	0.970
Foot	0.988	0.916	0.897	0.959	0.912	0.935
**PORH**						
Ulcer	0.628	0.790	0.861	0.914	0.949	0.865
Ulcer edge	0.706	0.846	0.894	0.932	0.955	0.952
Toe	0.828	0.910	0.883	0.917	0.874	0.949
Foot	0.883	0.952	0.901	0.903	0.905	0.960
**Buerger’s test**						
Ulcer	0.932	0.954	0.918	0.865	0.913	0.851
Ulcer edge	0.912	0.945	0.894	0.953	0.869	0.923
Toe	0.970	0.978	0.962	0.931	0.951	0.937
Foot	0.980	0.958	0.973	0.980	0.953	0.952

Legend: The recordings were observed by assessors 1 and 2, and the algorithm (Alg). The Interclass Correlation Co-efficients (ICCs) were calculated for the three Timespans of Interest (Baseline perfusion, Post-Occlusive Reactive Hyperemia (PORH), and Buerger’s test) and the four Regions of Interest (ulcer, ulcer edge, toe, and foot). Note: *p* ≤ 0.001 for all ICCs.

## Supplementary Materials

The following are available online at https://www.mdpi.com/2075-4418/10/12/1054/s1. Three curated videos of diabetic feet showing the ROI repositioning during baseline, Post-Occlusive Reactive Hyperaemia Test, and Buerger’s test measurements.
